# Multiple formin proteins participate in glioblastoma migration

**DOI:** 10.1186/s12885-020-07211-7

**Published:** 2020-07-29

**Authors:** Vanina D. Heuser, Aida Kiviniemi, Laura Lehtinen, Sune Munthe, Bjarne Winther Kristensen, Jussi P. Posti, Jussi O. T. Sipilä, Ville Vuorinen, Olli Carpén, Maria Gardberg

**Affiliations:** 1 Laboratory Division, Department of Pathology, Turku University Hospital, Turku, Finland; 2 Institute of Biomedicine, University of Turku, Turku, Finland; 3 Department of Radiology, Turku University Hospital and University of Turku, Turku, Finland; 4 Department of Neurosurgery, Odense University Hospital, Odense, Denmark; 5 Department of Pathology and Department of Clinical Research, Odense University Hospital, Odense, Denmark; 6 Division of Clinical Neurosciences, Department of Neurosurgery and Turku Brain Injury Centre, Turku University Hospital, Turku, Finland; 7 Department of Clinical Neurosciences, University of Turku, Turku, Finland; 8 Department of Neurology, Siun sote, North Karelia Central Hospital, Joensuu, Finland; 9 Division of Clinical Neurosciences, Department of Neurology, Turku University Hospital, Turku, Finland; 10 Department of Neurology, University of Turku, Turku, Finland; 11 Division of Clinical Neurosciences, Department of Neurosurgery, Turku University Hospital, Turku, Finland; 12 Department of Pathology, Helsinki University Hospital and University of Helsinki, Helsinki, Finland

**Keywords:** Actin, Formin, Glioma, Glioblastoma, Migration, Spheroid, FHOD1, INF2, Knockdown, Immunohistochemistry, Outcome

## Abstract

**Background:**

The prognosis of glioblastoma remains poor, related to its diffuse spread within the brain. There is an ongoing search for molecular regulators of this particularly invasive behavior. One approach is to look for actin regulating proteins that might be targeted by future anti-cancer therapy. The formin family of proteins orchestrates rearrangement of the actin cytoskeleton in multiple cellular processes. Recently, the formin proteins mDia1 and mDia2 were shown to be expressed in glioblastoma in vitro, and their function could be modified by small molecule agonists. This finding implies that the formins could be future therapeutic targets in glioblastoma.

**Methods:**

In cell studies, we investigated the changes in expression of the 15 human formins in primary glioblastoma cells and commercially available glioblastoma cell lines during differentiation from spheroids to migrating cells using transcriptomic analysis and qRT-PCR. siRNA mediated knockdown of selected formins was performed to investigate whether their expression affects glioblastoma migration.

Using immunohistochemistry, we studied the expression of two formins, FHOD1 and INF2, in tissue samples from 93 IDH-wildtype glioblastomas. Associated clinicopathological parameters and follow-up data were utilized to test whether formin expression correlates with survival or has prognostic value.

**Results:**

We found that multiple formins were upregulated during migration. Knockdown of individual formins mDia1, mDia2, FHOD1 and INF2 significantly reduced migration in most studied cell lines. Among the studied formins, knockdown of INF2 generated the greatest reduction in motility in vitro. Using immunohistochemistry, we demonstrated expression of formin proteins FHOD1 and INF2 in glioblastoma tissues. Importantly, we found that moderate/high expression of INF2 was associated with significantly impaired prognosis.

**Conclusions:**

Formins FHOD1 and INF2 participate in glioblastoma cell migration. Moderate/high expression of INF2 in glioblastoma tissue is associated with worse outcome. Taken together, our in vitro and tissue studies suggest a pivotal role for INF2 in glioblastoma. When specific inhibiting compounds become available, INF2 could be a target in the search for novel glioblastoma therapies.

## Background

Glioblastoma is the most common primary brain tumor among adults. The prognosis is poor, mainly due to its widely infiltrative behavior. At the time of diagnosis, cancer cells are invariably present within the brain parenchyma outside the tumor, frequently as far as in the contralateral hemisphere [1]. Recent studies have shown that migrating cells infiltrating the brain express stem cell markers [2]. Glioblastoma cells that express stem cell markers are more resistant to radiotherapy and chemotherapy [3, 4]. Cells outside the tumor bulk may also be connected by microtubes, forming a network of infiltrative cells that contribute to this resistance to treatment [5, 6]. Taken together, these findings suggest that infiltrating cells may be even more treatment resistant than the tumor bulk. In order to target infiltrating cells, more knowledge about their cell biology is needed.

Remodeling of the actin cytoskeleton is crucial in cell migration, mediated by actin-regulating proteins that are active in different cellular locations. New actin filaments and networks are formed at the leading edge by actin nucleating and elongating factors while severing proteins depolymerize in other cellular locations. Bundling proteins are needed for the formation of contractile actin stress fibers [7]. The largest group of actin filament nucleating and elongating proteins is the formin family of proteins, which catalyze the formation of unbranched actin filaments for both physiological and cancer-associated processes. The 15 genes encoding formin proteins are differentially expressed in human tissue types [8]. Individual formins are generally inactive until activated by Rho GTPases or phosphorylation. They have diverse biochemical and functional properties in shaping actin filaments. Their common denominator is the presence of formin homology 1 (FH1) and formin homology 2 (FH2) domains, with capacity to recruit profilin-bound actin monomers and add them to growing actin filaments [9].

The aims of this study were, first, to investigate how the expression of formins is altered in migrating glioblastoma cells in an in vitro model, second, to evaluate the importance of selected formins for migration in functional studies, and third, to study the expression of FHOD1 (formin homology domain containing protein 1) and INF2 (inverted formin 2) in human glioblastoma specimens.

## Methods

### Patients and clinical data

Patient-derived cell lines at Turku University Hospital and the glioma tissue microarrays (TMAs) with patient follow-up data were established with permission from the Auria Biobank steering committee and the Hospital District of Southwest Finland. For cell culture, all patients had given a written Biobank consent statement. The samples for TMAs and associated data were obtained as described previously [10], in accordance with the Finnish Biobank Act (688/2012) which does not require a separate informed consent from individual patients.

### Transcriptomic analysis

As a pilot study, we utilized data from a transcriptomic analysis of migrating primary glioblastoma cells compared to cells grown as spheroids from a previous study [2]. To summarize the experiment the data originated from, five cell lines (T111, T113, T78, T86 and T87) were established from glioblastoma tumor samples and grown in serum free conditions as spheroids. Spheroids had been plated in a semisolid matrix where part of the cells differentiated into elongated migrating cells. The migrating cells had been mechanically separated from non-migrating cells. RNA from migrating cells and free floating spheroids had been subjected to array-based transcriptomic analysis (Affymetrix 133 Plus 2.0 microarray). From the normalized data, we extracted the expression data of all 15 formin genes Formin 1 (FMN1), Formin 2 (FMN2), Disheveled-associated activator of morphogenesis 1 (DAAM1), Disheveled-associated activator of morphogenesis 2 (DAAM2), diaphanous related formin 1 (mDia1), diaphanous related formin 3 (mDia2), diaphanous related formin 2 (mDia3), FHOD1, Formin Homology 2 Domain Containing 3 (FHOD3), Formin-like protein 1 (FMNL1), Formin-like protein 2 (FMNL2); Formin-like protein 3 (FMNL3), Delphilin or delta 2-interacting protein 1 (GRID2IP), Inverted formin 1 (INF1), and INF2 and investigated their expression as fold-change in migrating cells compared to spheroid cells. Over 2 fold-change was considered upregulation in migrating cells.

### Cell culture

The commercially available glioblastoma human cell lines U87, U138 and T98 were from American Type Culture Collection (Manassas, VA, USA; catalogue numbers HTB-14, HTB-16 and CRL-1690, respectively). U138 and T98 were maintained in DMEM supplemented with 10% fetal bovine serum (FBS), 5 mM ultraglutamine and 100 U/ml penicillin-streptomycin (Gibco, CA, USA), while U87 was kept in spheroid medium (DMEM/F12, 1% B27, 100 U/ml penicillin-streptomycin (Gibco), 40 ng/ml fibroblast growth factor-2 (FGF-2; Gibco), and 40 ng/ml epidermal growth factor (EGF; Invitrogen, CA, USA).

Primary glioblastoma cell cultures established from glioblastoma samples at Turku University Hospital were named University of Turku Glioblastoma (UTGB). Following surgical resection, approximately 1–2 g of tumor tissue was collected and cultured as described by Rustenhoven et al. [11] From 10 tumor samples, 3 cell lines were established and tested negative for mycoplasma. The cell lines had the same mutations as the original tumor as tested by a 20 gene NGS panel designed for glioma diagnostics [12]. One cell line did not form spheroids, and was omitted from further studies. The clinicopathological characteristics of the two remaining primary cell lines UTGB4 and UTGB7 are described in Supplemental Table 1. In addition to these primary cells lines, we obtained T78, T86, and T87 primary glioblastoma cell lines that were included in the transcriptomics analysis [2]. All cell lines were repeatedly tested for mycoplasma contamination, and remained negative.

### Spheroid formation

Spontaneous spheroid formation was observed for U87, T86 and UTGB7 cells 3 to 6 days after plating single cells in spheroid medium. For all the other cell lines, spheroid formation was achieved using the hanging-drop technique for 48 h in 6 μl spheroid medium (UTGB4, 4 × 10^3^ cells/drop; T78 and T87, 2 × 10^3^ cells/drop) or DMEM 10% FBS (T98 and U138, 2 × 10^3^ cells/drop). Only spheroids with the diameter of 200–600 μm were used in the experiments.

### Cell migration

In order to study if and which formins are up or downregulated in migrating cells, spheroids were allowed to adhere in Geltrex (hESC-Qualified, Ready-To-Use, Reduced Growth Factor Basement Membrane Matrix, Gibco) precoated 6-well plate (30–40 spheroids/well) during 24 h. Using a microscope, attached spheroids were separated with the help of a needle and recovered from the supernatant. Migrated cells were detached with trypsin-EDTA solution (Sigma-Aldrich), and pelleted by centrifugation.

### qRT-PCR

Total RNA from different fractions was extracted using NucleoSpin RNA/Protein Kit (Macherey-Nagel, PA, USA) according to the manufacturer’s protocol and processed to cDNA with SensiFast cDNA Synthesis Kit (Bioline, OH, USA). TaqMan qRT-PCR was performed with a QuantStudio 12 K Flex Real-Time PCR System (Turku Centre for Biotechnology) using specific primers (Supplement 1) and quantitation was carried out with QuantStudio 12 K Flex software using the ΔΔCT method in order to calculate the relative fold gene expression. Three replicate samples were studied for detection of target mRNA expression and GAPDH was used as an endogenous control. The quantities were expressed as a fold change relative in migrating cells compared to immotile spheroids. The results are presented as means.

### Transfection with small interfering RNAs, spheroid migration assay and stainings

The mDia1, mDia2, FHOD1,and INF2 transcripts were knocked down individually in the commercial cell lines U87 and U138, and primary glioblastoma cell lines T86 and UTGB7 using 50 nM of SMARTpool small interfering RNA (siRNA) (Dharmacon Research, Boulder, CA). Non-targeting Pool siRNA was used as control. Cells attached as monolayer on Geltrex precoated 12-well plates were transfected using Dharmafect 4 transfection reagent (Dharmacon Research) according to the manufacturer’s instructions. The knockdown efficacy was examined 48 h after transfection by western blotting as described elsewhere [13]. Antibodies, dilutions and spheroid staining procedures are described in Supplement 1.

Forty-eight hours after siRNA treatment, cells were trypsinized and plated as drops for spheroid formation for another 48 h. To analyze migration spheroids were plated in Geltrex precoated 96-well Image Lock plate (Essen Bioscience, Ann Arbor, MI) and imaged every 2 h using the Incucyte S3 incubator system (Essen Bioscience). Areas of migrated cells and spheroids were used to indicate migration fold increase related to time point 1. The experiment was repeated at least 3 times, with a total of 25–50 spheroids per cell line for each siRNA treatment. Differences in migration from spheroids were analyzed using Student t-test. Two-tailed *p* values ≤0.05 were regarded as significant.

### Immunohistochemistry of glioblastoma samples

3.5 um sections from TMA:s composed of 1.5 mm cores of glioma samples were obtained from the Auria Biobank. The cohort includes diffuse glioma samples and has been previously described in detail [10]. It also includes relevant clinicopathological parameters and follow-up data. From this cohort, we analyzed the 93 samples that had an integrated diagnosis of glioblastoma, IDH-wildtype according to the WHO 2016 classification [14]. The slides were stained by the streptavidin-peroxidase method; using a Labvision staining device (Thermo Fisher Scientific, Fremont, CA) with a Bright Vision Poly-HRP-anti-mouse/rabbit kit (Immunologic, Duiven, the Netherlands) as described previously [15]. Briefly, antigen retrieval for INF2 staining was carried out by microwaving the slides in a pH 9 buffer. Primary antibody dilutions used were 1:75 for anti-FHOD1 (Sigma-Aldrich, St Louis, MA; catalogue number HPA024468) and 1:500 for INF2 (Proteintech, Chicago, IL; catalogue number 20466–1-AP). Furthermore, 10 whole slide samples were evaluated to compare FHOD1 and INF2 expression in tumor bulk to areas of diffuse tumor cell infiltration in brain tissue. For this, 10 cases with immunohistochemically detected p53 positivity indicating TP53 mutation and representative areas of solid tumor and diffuse infiltration were selected. The p53 stainings were performed using Ventana reagents and a Ventana Benchmark ULTRA autostainer (Ventana Medical Systems, Tucson, AZ).

Scoring was performed by two pathologists blinded to clinical data (MG and OC), with 0 standing for negative, 1 for weak, 2 for moderate and 3 for strong staining. Representative images of staining categories are presented in Fig. 3.

For survival analyses, both FHOD1 and INF2 staining scores were dichotomized into low expression (score 0 or 1) or high expression (score 2 or 3). Kaplan-Meier with log-rank test and univariate and multivariate Cox proportional hazards model were performed to assess survival. Multivariate Cox regression was analyzed using adjustments for age, pre-operative Karnofsky Performance Scale (KPS), resection type, and post-operative adjuvant treatment. Overall survival (OS) was defined as the time from surgical resection to death or end of follow-up. Progression-free survival (PFS) was a composite end-point defined as the time from surgical resection to the first tumor progression indicated by re-resection, start of a new treatment regimen, death, or end of follow-up. Survival analyses were conducted using IBM SPSS Statistics version 25.0 for Mac (IBM Corp., Armonk, NY).

## Results

### INF2 and FHOD1 are upregulated in migrating glioblastoma cells

To investigate the expression profiles of formins in glioblastoma, we studied the mRNA expression of all 15 formin genes in five different patient-derived glioblastoma cell lines. The cell lines had been subjected to a spheroid migration experiment, followed by transcriptomic analysis of migrating cells and spheroids [2, 16]. The results are presented in Table 1, as fold-change in migrating cells as compared to non-migrating cells. None of the formins was either up- or downregulated in every cell line. However, seven formins were upregulated upon migration in one or several cell lines. Of these, FMN2, DAAM1, DAAM2, mDia3 and FMNL2 were upregulated in single cell lines. Notably, INF2 and FHOD1 were widely upregulated 8–141-fold, in three out of five cell lines. All five cell lines upregulated either INF2 or FHOD1, or both.

**Table 1:**
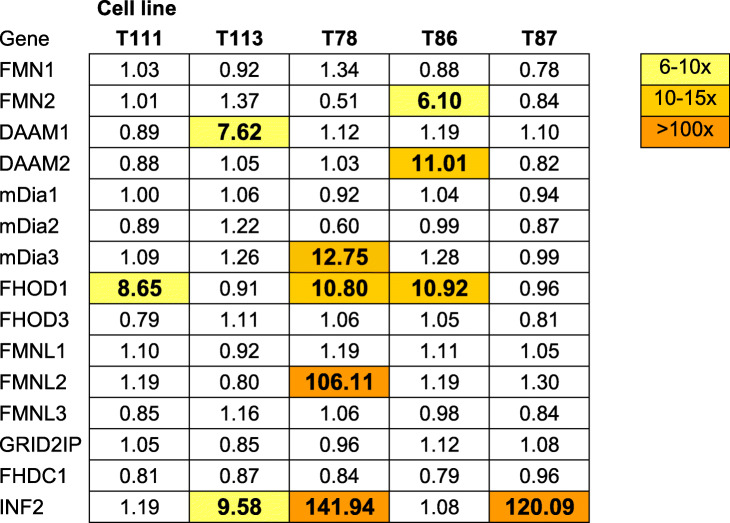
Transcriptomic analysis of formin expression in migrating glioblastoma cells compared to cells grown as spheroids. Results are presented as fold-change in five different primary glioblastoma cell lines T111, T113, T78, T86 and T87. In individual cell lines, over 2-fold change was observed for seven formins (highlighted as yellow to orange as heatmap)

Next, we wanted to test whether formin upregulation during spheroid migration could be seen in an expanded set of glioblastoma cell lines by using qRT-PCR. For this experiment, we included primary glioblastoma cell lines T78, T86 and T87 (present in transcriptomic analysis) and UTGB4 as well as UTGB7, which had been established at Turku University Hospital. For comparison, we also included commercially available glioblastoma cell lines U87, T98 and U138. This time, we compared the mRNA expression profiles of formins between migrating cells and spheroids plated on Geltrex (all attached). The results varied between the cell lines, with fold-change of individual formins varying from 0.1–791.4 (Table 2). The most commonly upregulated formin was mDia1 (5/8 cell lines), followed by INF2 (4/8 cells lines). FMN2, FHOD1, FHOD3, and FMNL1 were found upregulated in 3/8 cell lines. Six formins were upregulated in 1–2 cell lines each. Cell lines T98 and U138 upregulated multiple formins. The primary cell lines T78, T86 and T87 had increased expression of FHOD1 (2/3) and INF2 (3/3). Even though several increases of FHOD1 and INF2 expression were consistent between microarray and qRT-PCR results, they were not of similar magnitude. With qRT-PCR, the seen upregulation was mainly 2-fold.

**Table 2:**
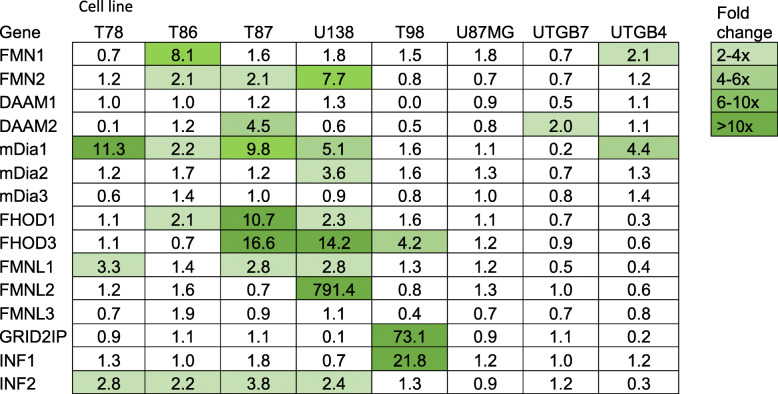
mRNA expression in migrating glioblastoma cells compared to spheroids. Results are presented as fold-change in five different primary glioblastoma cell lines and three commercially available cell lines. In individual cell lines, over 2-fold change was observed for 12 formins shown as different shades of green as heatmap

### Formin knockdown reduces glioblastoma cell migration

**Fig. 1:**
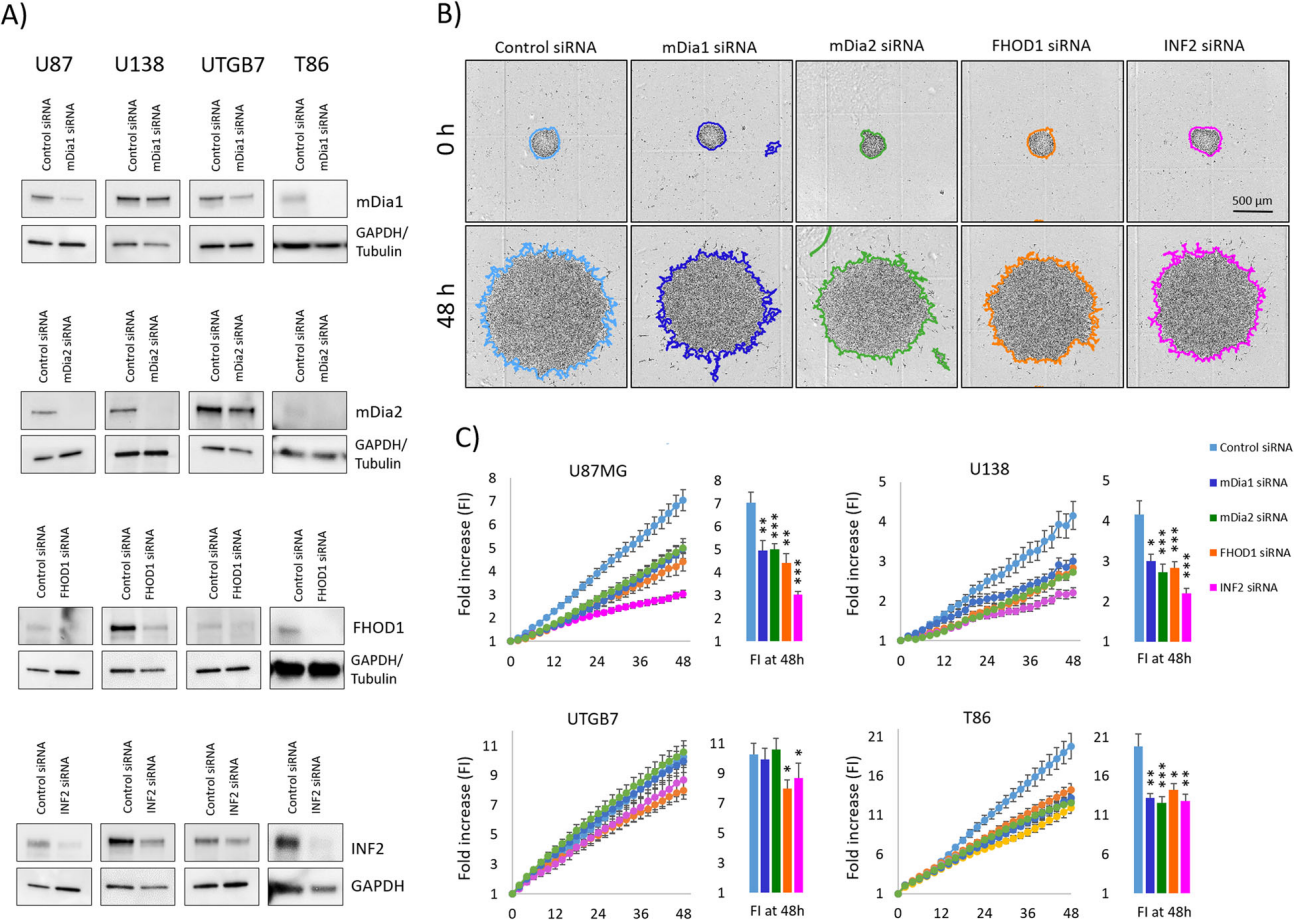
Knockdown of mDia1, mDia2, FHOD1, and INF2 expression in U87, U138, and primary glioblastoma cell lines UTGB7 and T86. **a** The knockdown efficacy was examined 48 h after transfection by immunoblotting. GAPDH or tubulin was used as a control for protein loading. Representative immunoblots have been cropped from repeated experiments to save space. The full-length blots are presented in Supplement 2. **b**) Representative images from spheroid migration experiment using the T86 primary cell line. Timepoints 0 and 48 h are shown. The edge of migrating cells is drawn. **c**) Plots showing fold increase of areas (FI) as a function of time (images were taken every 2 h). Bar graphs highlight the FI in individual treatment groups at 48 h. Asterisks indicate significant reduction of migration: * *p* ≤ 0.05, ** *p* ≤ 0.01, and *** *p* ≤ 0.001

The results from the spheroid migration experiments indicate that cells differentiating from spheroids to elongated migrating cells upregulate only selected formins. Therefore, we decided to study whether the basal expression of formins FHOD1 and INF2 is necessary for migration, using siRNA mediated knockdown. As formins mDia1 and mDia2 have earlier been shown to participate in glioblastoma migration [17–19], we decided to include knockdown of these formins in our experiments. The expression of the formins was individually knocked down in glioblastoma cell lines U87, U138, T86, and UTGB7. Western blotting was used to check that knockdown was efficient (Fig. 1a). The targeted protein level was markedly reduced in a vast majority of the cell lines.

**Fig. 2:**
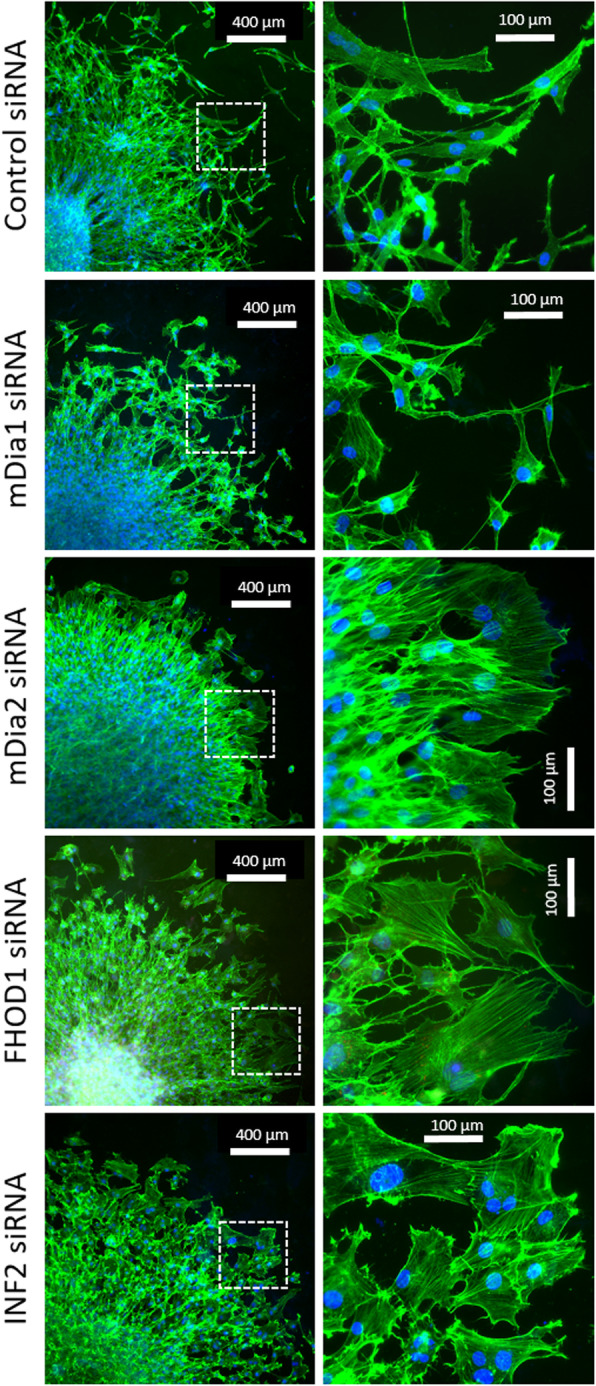
Representative pictures of U87 spheroids migration after treatment with different siRNAs. Spheroids were plated in Geltrex precoated coverslips and grown for 24 h before they were stained with phalloidin (green) and DAPI (blue) in order to visualize actin filaments and nuclei. Morphological changes can be seen after the knockdown of mDia2, FHOD1, and INF2

INF2 and FHOD1 knockdown reduced cell migration significantly in all four cell lines (15–44%) (Fig. 2a and b, Supplemental Table 2). The knockdown of mDia1 and mDia2 had a slighter and more variable effect, with statistically significant reduction of migration (by 12–30%) in cell lines U87, U138 and T86, but without significant effect in cell line UTGB7.

Cell morphology in migrating cells was notably altered by knockdown of formins. Similar changes were seen in all cell lines. Representative images of U87 spheroids are depicted in Fig. 2. INF2, FHOD1 and mDia2 knockdown resulted in spread cells with a more epithelial appearing phenotype. With mDia1 knockdown no apparent morphological change was observed as cells maintained their spindle-like morphology.

### High INF2 expression in clinical glioblastoma samples is associated with worse outcome

To establish whether the most interesting formins from the transcriptomics analysis, FHOD1 and INF2, are expressed as protein in human glioblastoma, we performed immunohistochemistry on human glioblastoma samples. The antibodies used for immunohistochemistry bind specifically to FHOD1 and INF2. The FHOD1 antibody used for immunohistochemistry has previously been validated by us [20]. The INF2 antibody used was the same as in Western Blotting, where it detected a major band of expected size. The band was attenuated when cells were treated with INF2 siRNA, indicating specific binding (see Fig. 1a).

**Fig. 3:**
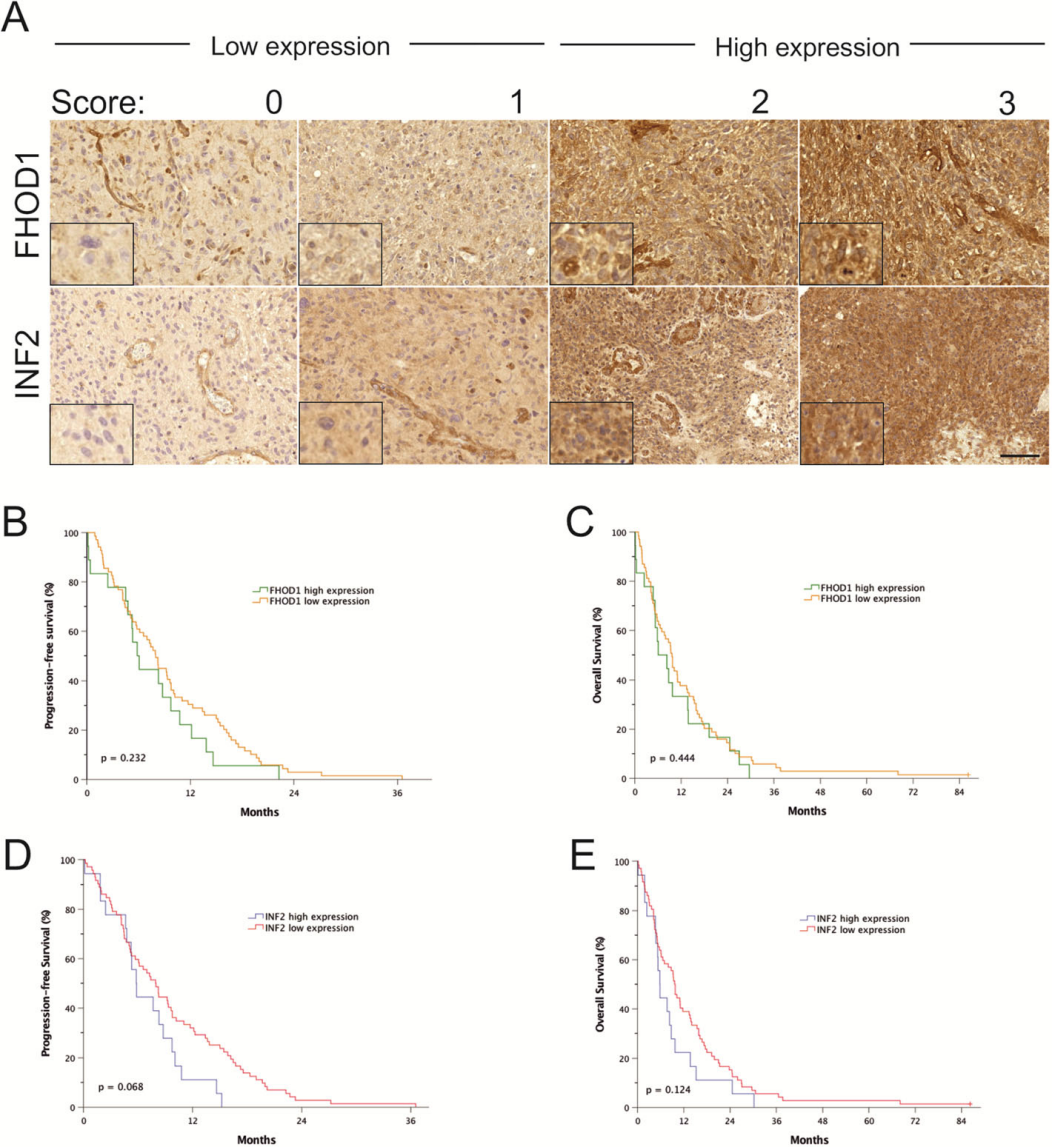
Immunohistochemistry for FHOD1 or INF2 and progression free or overall survival in glioblastoma according to expression level. **a**) Immunohistochemistry for FHOD1 (upper panel) and INF2 (lower panel) was performed in tissue from 93 glioblastomas. Staining was scored in glioblastoma cells as negative = 0, low = 1, moderate = 2, strong = 3. Note that endothelial cells are clearly FHOD1 and INF2 positive in all categories. Dichotomization was performed by grouping scores 0 and 1 (low expression), and 2 and 3 (high expression). Scale bar: 100 μm. Insets present details with higher magnification. **b**, **c**) Progression-free and overall survival according to FHOD1 expression. **d**, **e**) Progression-free and overall survival according to INF2 expression

In 93 samples of IDH-wildtype glioblastomas, FHOD1 and INF2 expression (scores 1–3) could be detected in 84 and 46% of cases, respectively. Positive staining was diffusely cytoplasmic (Fig. 3a).

After dichotomization, 18 glioblastomas showed high expression of FHOD1 (score 2 or 3) and 69 low expression (score 0 or 1). FHOD1 staining was unsuccessful for six specimens. For INF2, 18 glioblastomas showed high expression, 72 low expression, and 3 specimens were missing.

Survival plots (Fig. 3b-e) show no significant difference in progression-free survival (PFS) or overall survival (OS) in glioblastomas according to FHOD1 and INF2 expression, although slightly inferior median PFS and OS was noted in the INF2 high expression groups (Supplemental Table 3; *p* values 0.068 and 0.124, respectively).

In univariate Cox regression analysis, no significant difference in PFS or OS was observed based on the expression level of FHOD1 or INF2. Glioblastoma outcome is highly influenced by several clinicopathological parameters. Therefore we proceeded with multivariate analysis. When adjusted for age, pre-operative KPS, resection type, and post-operative treatment, high expression of INF2 was an independent predictor for worse PFS and OS (Table 3). No difference in survival was observed related to FHOD1 expression.

**Table 3:** Multivariate Cox regression model for PFS and OS

Variable	PFS			OS		
	HR	95% CI	***p***-value	HR	95% CI	***p***-value
Age	1.003	0.98–1.03	0.833	1.002	0.98–1.03	0.853
FHOD1						
Low expression	1			1		
High expression	1.19	0.57–2.46	0.643	1.0	0.50–2.02	1.000
INF2						
Low expression	1			1		
High expression	2.33	1.05–5.18	0.038*	2.22	1.04–4.77	0.041*
Pre-operative KPS			0.145			0.019*
100	1			1		
90	0.81	0.30–2.12	0.674	0.39	0.15–1.07	0.068
80	1.25	0.48–3.31	0.648	0.75	0.28–2.02	0.568
70	1.29	0.48–3.46	0.619	1.12	0.42–2.98	0.82
60	2.05	0.70–6.02	0.191	1.80	0.62–5.23	0.281
50	1.76	0.56–5.52	0.335	1.38	0.44–4.33	0.583
40	1.55	0.41–5.79	0.516	1.03	0.28–3.88	0.962
30	6.72	1.23–36.85	0.028	5.40	0.99–29.6	0.052
20	1.55	0.30–7.99	0.603	1.51	0.29–7.76	0.623
10	62.8	3.56–1109.3	0.005	39.8	2.28–693.2	0.012
Resection						
Gross total	1			1		
Subtotal	2.34	1.17–4.69	0.016*	1.69	0.83–3.43	0.151
Post-operative treatment			< 0.001**			< 0.001**
RT + TMZ	1			1		
RT only	2.34	1.22–4.48	0.010	2.47	1.31–4.67	0.005
None	7.34	2.79–19.29	< 0.001	7.97	3.00–21.15	< 0.001

*Abbreviations*: *KPS* Karnofsky Performance Scale, *RT* Radiotherapy, *TMZ* Temozolomide

**Fig. 4:**
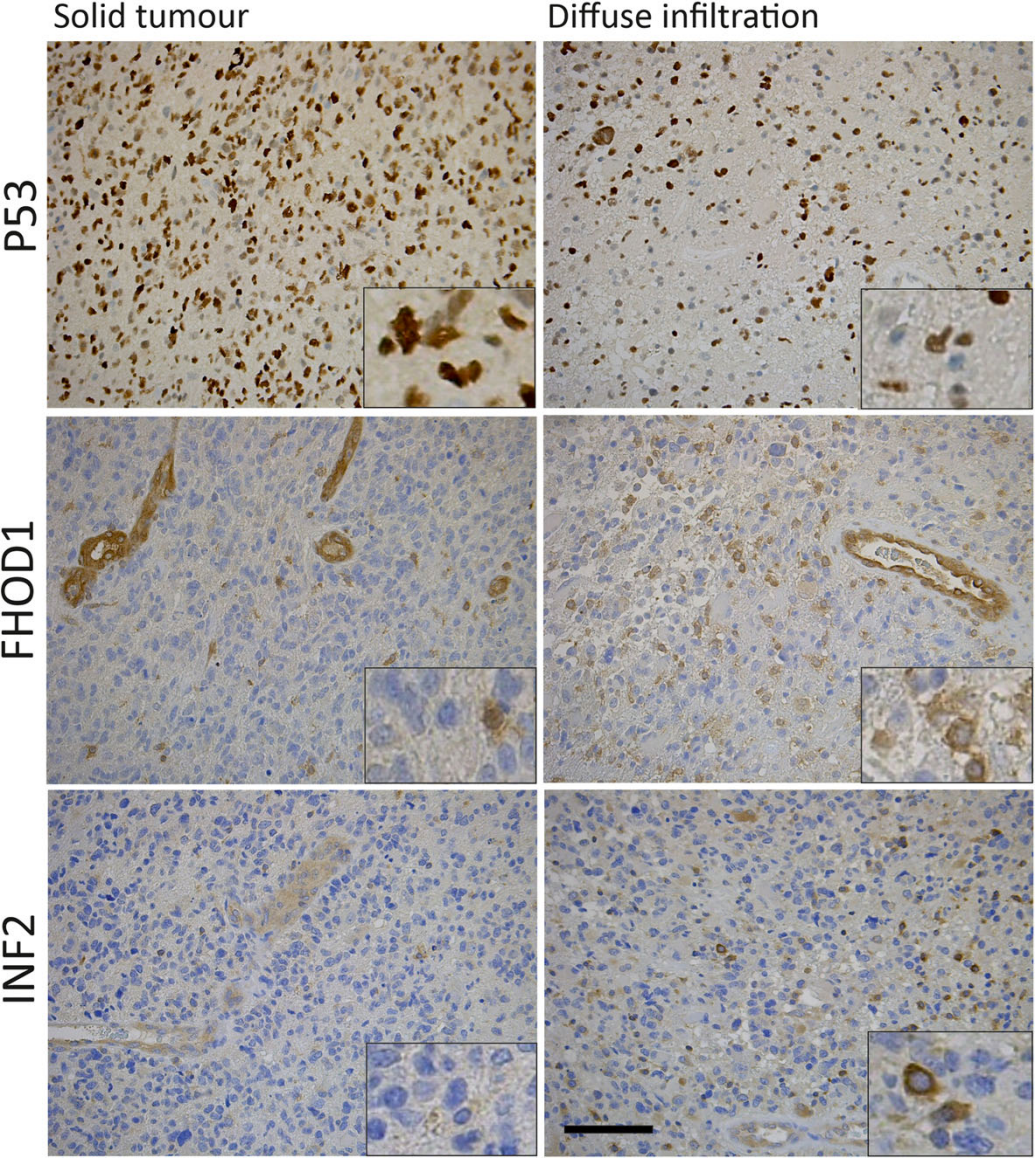
Immunohistochemistry for FHOD1 and INF2 in TP53 mutant glioblastoma, comparing solid areas with infiltrating areas. The top micrographs show p53 staining in solid tumour (left) and area of diffuse infiltration (right). Tumour cells have positive staining nuclei, while non-neoplastic cells remain negative. FHOD1 staining in the corresponding tumour areas show negative staining in a vast majority of tumour cells and positive staining in tumour vessel walls. The infiltrating area of the same glioblastoma shows moderate/high FHOD1 expression in 20% of infiltrating tumour cells. The lower micrographs show INF2 staining in the same glioblastoma. The solid tumour is virtually INF2 negative, except for tumour vessels (lower left). The infiltrating area of the same tumour shows moderate/high expression in 20% of tumour cells (lower right). Insets show details with higher magnification

Expression of formins in TMA samples from the center of glioblastomas may be different from expression in glioblastoma cells infiltrating into the surrounding brain parenchyma. To evaluate whether FHOD1 or INF2 expression is altered in areas of diffuse infiltration as compared to solid tumour areas, we studied 10 representative glioblastoma samples as whole slides. The cases had immunohistochemically detected TP53 mutations, which made it possible for us to firmly differentiate infiltrating tumor cells from gliosis. In these samples, five glioblastomas were found to have diffusely infiltrating cells expressing moderate/high FHOD1 and/or INF2 in more than 20% of tumour cells, while the solid tumour showed absent/low expression (Fig. 4). In five glioblastomas, the expression of the formins remained unchanged when comparing infiltrating cells and solid tumour areas. This result suggests that FHOD1 and INF2 expression is not necessarily uniform, but can be higher in infiltrating cells than in tumour bulk.

## Discussion

Glioblastoma remains a challenging disease to treat. Although some progress has been made with surgical techniques and combining chemotherapy with radiation, the wide spread tumour cells combined with resistance to treatment still signifies dismal prognosis. For a breakthrough in glioblastoma treatment, infiltrative cells within the brain parenchyma should be targeted. Therefore, effort should be made to characterize the molecular machinery that defines their behavior. Our approach is to study proteins that regulate the actin cytoskeleton altering cell shape, adhesion and further directing membrane protrusion force to the leading edge. The actin cytoskeleton and its regulators could be valid targets for future therapy.

In this study, we utilized the ability of glioblastoma cell spheroids to differentiate into elongated migrating cells as an in vitro model for glioblastoma cell migration. Herein we report that formin mRNA upregulation in this event is generally minor, with variation from one cell line to another.

We found considerable difference in magnitude of alterations using a transcriptomic array compared to qRT-PCR. This difference can be attributable to methodological differences and possibly differences in laboratory conditions. We were able to validate that slight upregulation of formins INF2, FHOD1, FHOD3 and mDia1 occurs, which could have some relevance for migration. However the basal expression of INF2 and FHOD1 may be as important. Both INF2 and FHOD1 have earlier been found to participate in migration or invasion and to be upregulated in clinical cancer tissues in basal-like breast cancer [15], and FHOD1 has additionally been found expressed in oral squamous cell carcinoma and melanoma, also with participation in migration/invasion in vitro [13, 20].

Using a knockdown approach, we could for the first time show that the expression of INF2 and FHOD1 is necessary for efficient migration of glioblastoma cells. This effect was found both in commercial cell lines (U87 and U138) and in a primary glioblastoma cell lines (T86 and UTGB7). Previous studies that have focused on other formins than INF2 and FHOD1 have also indicated that individual formins participate in glioblastoma cell migration/invasion. First, a study on formin FHOD3 showed that a rapid form of migration induced by laminin tracks was dependent on FHOD3. This was indicated in rat C6 glioma cells and a primary glioma cell line [21]. Also mDia formins have been shown to participate in glioblastoma cell migration. mDia activities can be altered by small molecules, causing either agonism or antagonism. Of these, agonism has been shown to be more effective in glioblastoma cell line U87 [18]. Very recently, Pettee et al. explored the feasibility of mDia agonism as an anti-invasion strategy in patient-derived high-grade glioma cells. They found that the molecule IMM2 efficiently reduced the single cell migration from the neurosphere core. IMM2 treatment caused a morphologic shift toward ameboid morphology, also inhibiting the formation of microtubes [19]. Microtubes are recently found cellular extensions in glioblastoma, that keep glioblastoma cells connected as a network while migrating [5]. Inhibiting the formation of microtubes prolongs survival in a mouse model [6]. Although we did not look for changes in microtubes, our findings from mDia1 and mDia2 knockdowns were in line with previous studies. Migration was reduced, confirming effects seen by others [18]. This was additionally accompanied by a shift in morphology with mDia2 knockdown. Migrating glioblastoma cells lost extensions and mesenchymal morphology, similar to findings reported with IMM2 treatment [19]. Compared to mDia1 and mDia2, we found that FHOD1 knockdown had similar effect on migration. Importantly, we found clearly larger reduction of migration upon knockdown of INF2, suggesting that modification of its activity could be a valid target for treatment. To our knowledge there are currently no available specific inhibitors/agonists for this formin.

Using immunohistochemistry, we discovered that formins FHOD1 and INF2 are frequently expressed in glioblastoma tissue. The level of FHOD1 expression did not have prognostic significance, but in multivariate analysis cases with moderate/high expression of INF2 had a worse prognosis than those with absent/low expression of INF2. This is to the best of our knowledge the first report on formin protein expression in a large set of human glioblastoma samples. We have in previous studies found that FHOD1 is not expressed in brain parenchyma, although it is present in endothelial cells in the brain [20]. According to the human protein atlas (www.proteinatlas.org), INF2 expression is low in glial cells [22]. Therefore, the moderate/high expression of FHOD1 and INF2 in a subset of glioblastomas can be considered as overexpression. However, all glioblastomas did not express immunohistochemically detectable amounts of these formins, which suggests that their expression is not crucial for glioblastoma invasion. It seems likely the expression profiles of actin regulating proteins vary from case to other. In other words, the search for actin-targeting treatment may require a personalized approach.

## Conclusions

Our results indicate that individual formins are upregulated as primary glioblastoma cells differentiate from an immobile spheroid mass to elongated migrating cells. Contrary to our hypothesis, we could not identify a single formin that was upregulated in all cell lines. Instead, primary glioblastoma cell lines seem to have individual profiles in formin upregulation. This confirms that exploring molecular mechanisms in glioblastoma requires more than one or two cell lines before generalizations can be made. Although we found that knockdown of several formins reduced migration, the greatest effect was seen upon INF2 knockdown. Furthermore, moderate/high expression of INF2 in glioblastoma tissue was associated with worse outcome. Taken together, our in vitro and tissue studies suggest a pivotal role for INF2 in glioblastoma. When specific inhibiting compounds become available, INF2 could be a target in the search for novel glioblastoma therapies.

## Supplementary information

**Additional file 1.**

**Additional file 2.**

**Additional file 3.**

**Additional file 4.**

**Additional file 5.**

## Data Availability

The datasets generated and/or analyzed during the current study are available from the corresponding author on reasonable request. The data from transcriptomic analysis has been uploaded to GEO Datasets by Munthe et al. [2], accession number GSE76018.

## Abbreviations

mDia1: Diaphanous related formin 1
mDia2: Diaphanous related formin 3
qRT-PCR: Quantitative real-time polymerase chain reaction
siRNA: Small interfering RNA
FHOD1: Formin homology domain containing protein 1
INF2: Inverted formin 2
IDH: Isocitrate dehydrogenase
FH1: Formin homology 1
FH2: Formin homology 2
TMA: Tissue microarray
FMN1: Formin 1
FMN2: Formin 2
DAAM1: Disheveled-associated activator of morphogenesis 1
DAAM2: Disheveled-associated activator of morphogenesis 2
mDia3: Diaphanous related formin 2
FHOD3: Formin Homology 2 Domain Containing 3
FMNL1: Formin-like protein 1
FMNL2: Formin-like protein 2
FMNL3: Formin-like protein 3
GRID2IP: Delphilin or delta 2-interacting protein 1
INF1: Inverted formin 1
KPS: Karnofsky performance scale
PFS: Progression free survival
OS: Overall survival
