# Supplementary material for: Multiple formin proteins participate in glioblastoma migration
Source: BMC Cancer. 2020 Jul 29;20:710. doi: 10.1186/s12885-020-07211-7 (PMC7391617; doi:10.1186/s12885-020-07211-7)
Supplement: Supplementary file 1 — Additional file 1. [file 12885_2020_7211_MOESM1_ESM.docx]

**Supplement 1.** Specifications for methods used in cell studies.

Gene specific primers for qRT-PCR:

| Formin | forward 5′-3′ | reverse 5′-3′ |
| --- | --- | --- |
| FMN1 | cccatgtgagacacactgct | aagtgatgaggtgatgctgaca |
| FMN2 | ggatgttgacagcaaagaagg | tccgggagcaaaacttctc |
| DAAM1 | ggagctacaagttggcctga | tccttctctaaagccagcaga |
| DAAM2 | gatggtgcggaatgaggat | cactcttggtgtcgatgtgg |
| mDia1 | cagtcaggggcagcattc | cactgttcttggacaccttgg |
| mDia2 | gcgggaaaaggacttcagtat | ggtgagatctgtcggcttct |
| mDia3 | ccaccaaacgtgagatggtt | ctgtttttcagcccaccag |
| FHOD1 | cctcagctgacacctccag | cagcgcaacctgcttctc |
| FHOD3 | ggccaggttggaaaggtc | tctgctgccagtgactcttg |
| FMNL1 | ctttgcccagtgctctgtc | tggacccttgctgaggtct |
| FMNL2 | ttcctgtctttgtccggttt | tcctgctttttcctcagctc |
| FMNL3 | cctctgctgggcatgtgt | atggccagtgactcttttcc |
| GRID2IP | cgggcttcaagatcaacttt | gtggacttcccatccactgt |
| INF1 | gcatcatgttcagaagactgcta | tgtcctgacaaacagcaagtg |
| INF2 | tcgagtacatcctcagcaacc | atcacgttggatgtgtccag |
| GAPDH | acccactcctccacctttga | ttgctgtagccaaattcgttgt |

**Antibodies and dilutions used in Western Blotting**

The rabbit anti-human FHOD1, mDia1 (both from Sigma-Aldrich), INF2 (Proteintech, Rosemont, IL), and mDia2 (Abcam) antibodies were used at a 1:1000 dilution. Rabbit polyclonal GAPDH - HRP conjugated (Abcam) or α Tubulin mouse monoclonal (B-5-1-2, Invitrogen) antibody was 1:5000 as control for protein loading. The secondary antibodies were HRP-conjugated swine anti-rabbit and HRP-conjugated rabbit anti-mouse immunoglobulins (1:3000, Dako, Glostrup, Denmark) diluted in block solution. Membranes were washed three times with TBST between the different steps.

**Spheroid stainings**

Spheroids were plated in Geltrex precoated coverslips (13 mm) and grown for 24 hours. Next, the cells were fixed with 4% paraformaldehyde for 10 min at room temperature. The coverslips were washed with PBS and blocked with 5% BSA, 0.5% triton X-100 in PBS for 45 minutes. Next, the coverslips were incubated with Alexa Fluor 488- conjugated phalloidin (1:300, Invitrogen, Carlsbad, CA) for 1h and washed 3 times with PBS. The mounting media contained DAPI for staining nuclei (ProLong® Gold Antifade Mountant with DAPI, Life Technologies, UK). Images were taken with a Nikon Eclipse Ni fluorescence microscope and different channels were merged using ImageJ v1.52n software (http://rsbweb.nih.gov/ij/).
