# Supplementary figures and images for: Multiple formin proteins participate in glioblastoma migration
Source: BMC Cancer. 2020 Jul 29;20:710. doi: 10.1186/s12885-020-07211-7 (PMC7391617; doi:10.1186/s12885-020-07211-7)

Supplement 2: Original blots corresponding to Figure 1A

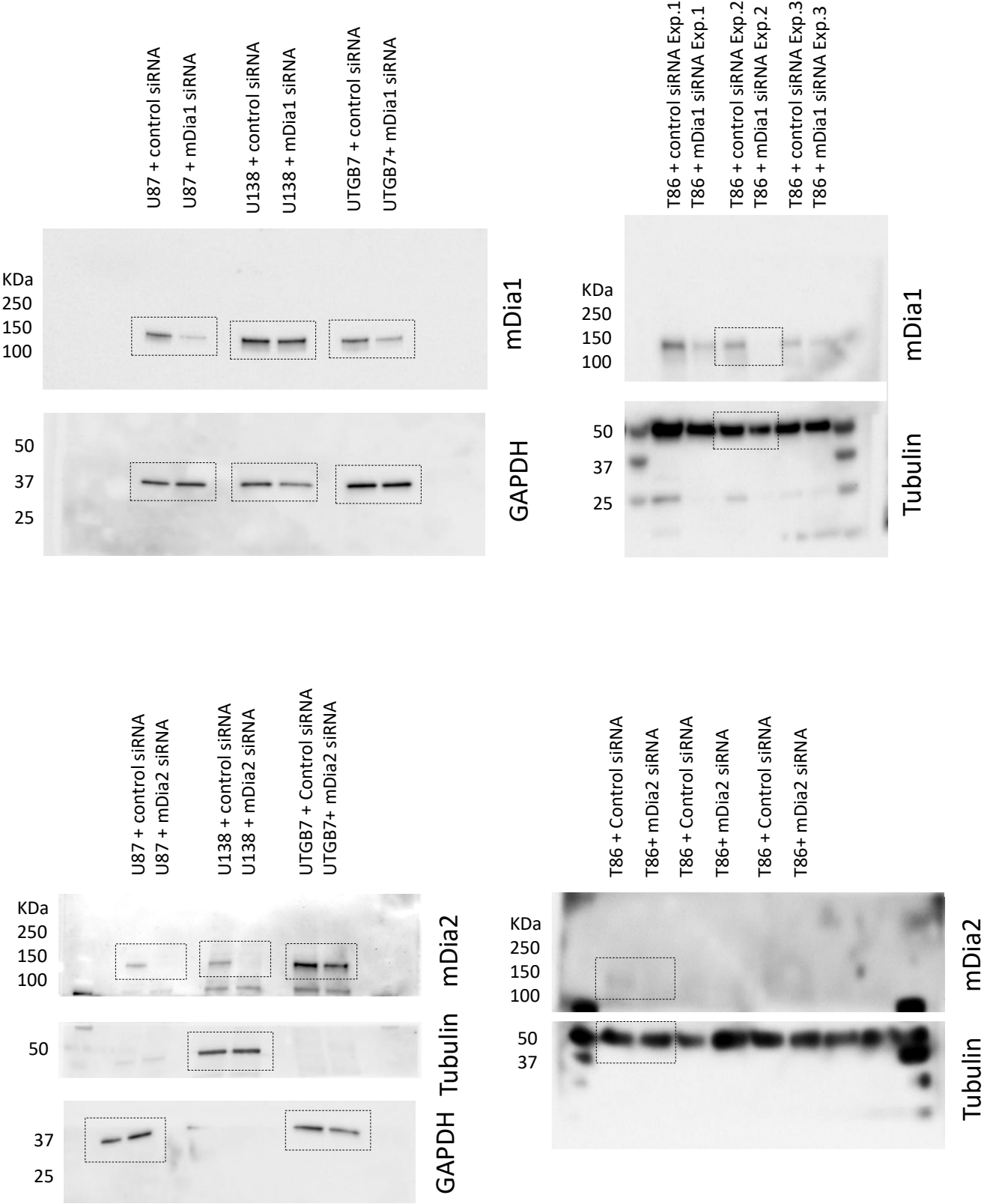

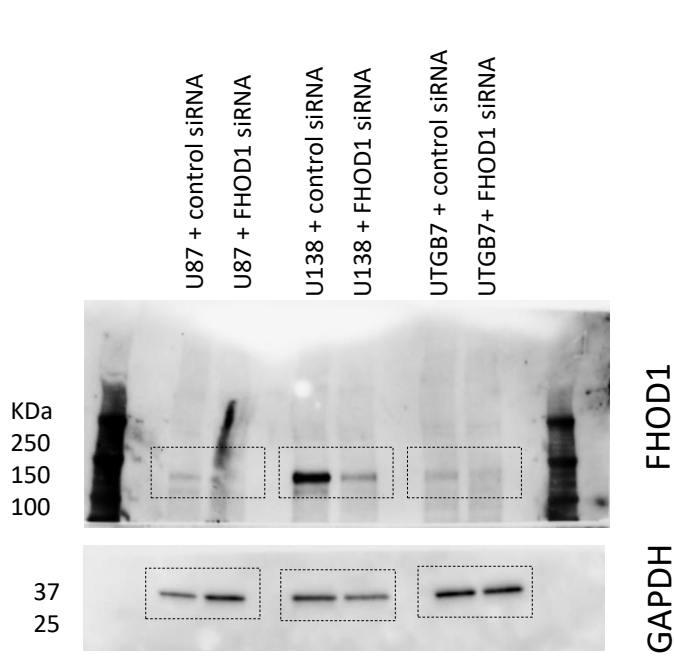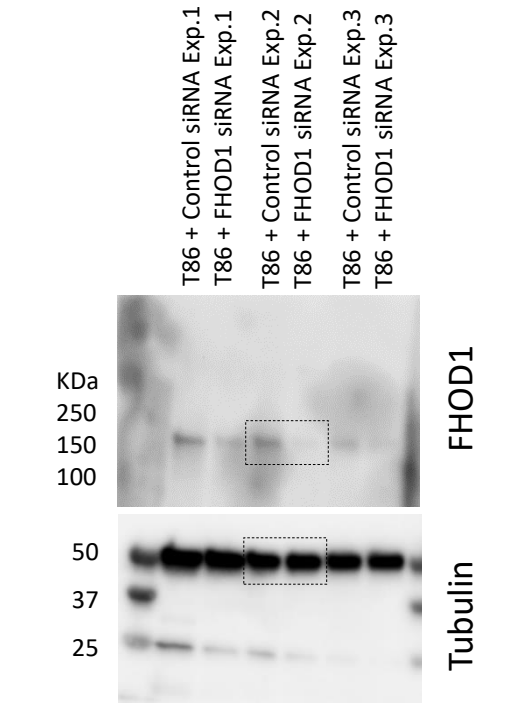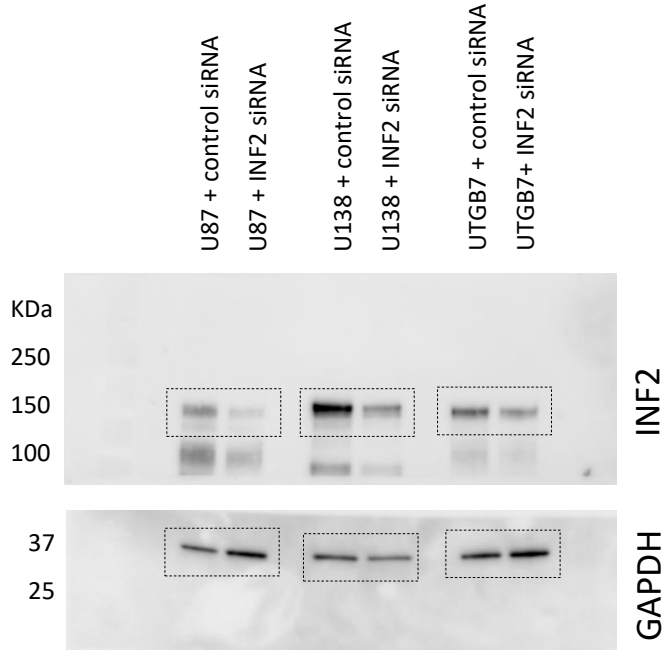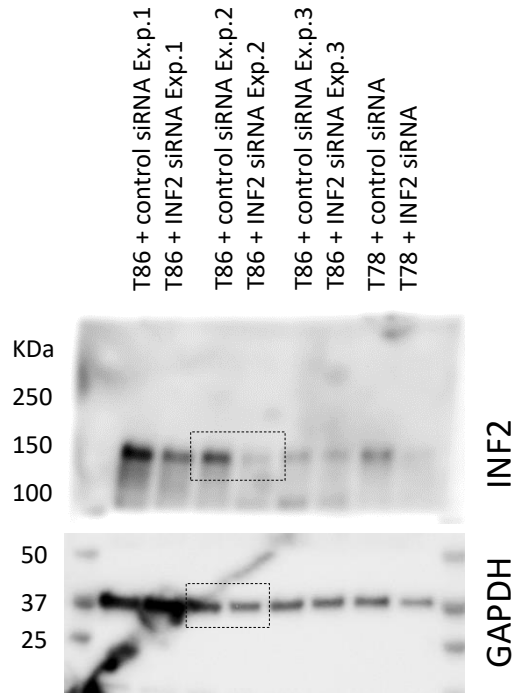

Supplement: Supplementary file 2 — Additional file 2. [file 12885_2020_7211_MOESM2_ESM.pdf]
