# Supplementary material for: Multiple formin proteins participate in glioblastoma migration
Source: BMC Cancer. 2020 Jul 29;20:710. doi: 10.1186/s12885-020-07211-7 (PMC7391617; doi:10.1186/s12885-020-07211-7)
Supplement: Supplementary file 3 — Additional file 3. [file 12885_2020_7211_MOESM3_ESM.pdf]

Supplemental table 1. Clinicopathological characteristics of patient-derived cell lines UTGB4 and UTGB7.

**Cell line**	**Age**	**Gender**	**Diagnosis**	**Tumor location**	**Proliferation (Ki-67 %)**	**Mutations in 20 gene NGS panel**[12]
UTGB4	86	female	Glioblastoma, IDH-wildtype	Left temporal lobe	60	TERT, EGFR, TP53
UTGB7	73	female	Glioblastoma, IDH-wildtype	Right temporal lobe	20	TERT. PIK3R1, PTEN, TP53
