# Supplementary material for: Multiple formin proteins participate in glioblastoma migration
Source: BMC Cancer. 2020 Jul 29;20:710. doi: 10.1186/s12885-020-07211-7 (PMC7391617; doi:10.1186/s12885-020-07211-7)
Supplement: Supplementary file 4 — Additional file 4. [file 12885_2020_7211_MOESM4_ESM.docx]

| **Supplemental table 2.** Spheroid migration results and statistics. | | | | |
| --- | --- | --- | --- | --- |
| **Cell line** | **Treatment group** | **N** | **Average area under the curve (±SE)** | **P *versus* control** |
| U87 | Control siRNA | 42 | 100 ± 5 |  |
|  | mDia1 siRNA | 51 | 81 ± 7 | **<0.001** |
|  | mDia2 siRNA | 49 | 72 ± 3 | **<0.0001** |
|  | FHOD1 siRNA | 37 | 72 ± 6 | **<0.001** |
|  | INF2 siRNA | 45 | 56 ± 3 | **<0.0001** |
|  |  |  |  |  |
| U138 | Control siRNA | 44 | 100 ± 5 |  |
|  | mDia1 siRNA | 46 | 88 ± 5 | **<0.05** |
|  | mDia2 siRNA | 25 | 70 ± 7 | **<0.0001** |
|  | FHOD1 siRNA | 50 | 77 ± 4 | **<0.0001** |
|  | INF2 siRNA | 48 | 70 ± 4 | **<0.0001** |
|  |  |  |  |  |
| UTGB7 | Control siRNA | 43 | 100 ± 5 |  |
|  | mDia1 siRNA | 25 | 86 ± 8 | 0.1518 |
|  | mDia2 siRNA | 38 | 103 ± 6 | 0.7124 |
|  | FHOD1 siRNA | 46 | 85 ± 4 | **<0.05** |
|  | INF2 siRNA | 29 | 80 ± 7 | **<0.05** |
|  |  |  |  |  |
| T86 | Control siRNA | 40 | 100 ± 13 |  |
|  | mDia1 siRNA | 35 | 71 ± 5 | **<0.0001** |
|  | mDia2 siRNA | 46 | 73 ± 8 | **<0.0001** |
|  | FHOD1 siRNA | 39 | 79 ± 7 | **<0.01** |
|  | INF2 siRNA | 40 | 73 ± 9 | **<0.001** |
