# Supplementary material for: Multiple formin proteins participate in glioblastoma migration
Source: BMC Cancer. 2020 Jul 29;20:710. doi: 10.1186/s12885-020-07211-7 (PMC7391617; doi:10.1186/s12885-020-07211-7)
Supplement: Supplementary file 5 — Additional file 5. [file 12885_2020_7211_MOESM5_ESM.pdf]

Supplemental table 3. Median survival according to formin expression.

	FHOD1			INF2		
	**Low expression** (score 0 or 1)	**High expression** (score 2 or 3)	p-value (Log-rank)	**Low expression** (score 0 or 1)	**High expression** (score 2 or 3)	p-value (Log-rank)
Median PFS (95% CI; months)	8.0 (6.8–9.1)	5.8 (4.2–7.4)	0.232	7.9 (6.2–9.7)	5.8 (4.7–6.9)	0.068
Median OS (95% CI; months)	9.6 (8.4–10.8)	6.1 (0.9–11.2)	0.444	9.6 (8.1–11.2)	5.8 (4.7–6.9)	0.124
